# 

**Published:** 1889-07-27

**Authors:** 


PRICE ONE PENNY.

No. 148, Vol. VI.—July 27th, 1889.

Registered at the General Post Office as a Newspaper.]

Per Annum, 6s. 6d., post free.

Monthly Parts, 6d.; post free 7½d.

Ditto per Annum, 7s. 6d., post free.

